# A new experimental model of peanut allergy in BALB/c and C57BL/6 mice

**DOI:** 10.3389/falgy.2025.1717401

**Published:** 2026-01-26

**Authors:** Marcos Felipe Andrade-Oliveira, Rafael Saavedra Langer, Sara Cândida Barbosa, Mariana Almeida Oliveira, Cecília Horta Ramalho-Pinto, Giovanna Caliman Camatta, Jamil Silvano de Oliveira, Igor de Campos Fontes, Fabiana Simão Machado, Carlos Delfin Chávez-Olórtegui, Tatiani Uceli Maioli, Denise Carmona Cara, Ana Maria Caetano Faria

**Affiliations:** 1 Departamento de Bioquímica e Imunologia, Instituto de Ciências Biológicas, Universidade Federal de Minas Gerais, Belo Horizonte, Brazil; 2 Departamento de Patologia, Instituto de Ciências Biológicas, Universidade Federal de Minas Gerais, Belo Horizonte, Brazil; 3 Departamento de Nutrição, Escola de Enfermagem, Universidade Federal de Minas Gerais, Belo Horizonte, Brazil; 4 Departamento de Morfologia, Instituto de Ciências Biológicas, Universidade Federal de Minas Gerais, Belo Horizonte, Brazil; 5 Instituto de Investigação em Doenças Infecciosas e Crônicas de Mucosas e Pele (INCT Mucosa e Pele), Belo Horizinte, Brazil

**Keywords:** food allergy, anaphylaxis, gut inflammation, IgE, peanut, secretory IgA

## Abstract

**Introduction:**

Food allergy protocols are highly variable. While it allows the exploration of multiple scientific questions, it limits the possibility to make general assumptions and point out specific features of allergy to different food allergens. Considering this, our research group has developed a standardized food allergy protocol that has allowed us to study egg, milk and shrimp allergy.

**Methodology:**

In this study, we used the same protocol to develop a model of peanut allergy. BALB/c and C57BL/6 mice were sensitized with a subcutaneous injection of 40mg of peanut protein extract (PPE) adsorbed in 3mg of aluminum hydroxide (Al(OH)_3_) on days 0 and 14. From day 21 to 35, as an oral challenge, sensitized and control mice received a whole peanut extract (WPE) as the only source of drinking solution, while naïve groups received water.

**Results:**

After oral challenge, BALB/c and C57BL/6 sensitized mice had higher levels of total and specific IgE in the serum, as well as specific IgG1, than control groups. BALB/c, but not C57BL/6, mice presented higher levels of anti-PPE secretory IgA (SIgA), and lower reactivity to fecal bacteria than control group. Sensitized mice from both strains presented positive cutaneous anaphylaxis reaction. Systemic anaphylaxis was more prominent in C57BL/6 mice, while increase in gastrointestinal transit time was more prominent in BALB/c mice. Lastly, jejunum of BALB/c and C57BL/6 sensitized mice presented higher eosinophil, intraepithelial lymphocyte, and goblet cell count, as well as smaller villi length, than control groups.

**Conclusion:**

Our standardized food allergy protocol enabled the development of a peanut allergy model in two different mouse strains, which not only allows the study of peanut allergy itself, but also its comparison to other allergies triggered by food proteins.

## 1 Introduction

Food allergy prevalence has been increasing over the last few decades, affecting 8%–11% of children and 10.8% of adults in developed countries (1–3). Lifestyle changes seem to be associated with this trend, which led to the formulation of the hygiene and dual allergen exposure hypotheses, for example (4). Although the exact immunological mechanisms that set off food sensitization are still unclear, they culminate in the generation of Th2 lymphocytes and IgE-producing B cells. IgE binds to the surface of mast cells and basophils via FcɛRI and, when cross-linked to food allergens, leads to the release of an array of inflammatory mediators (4). This is the basis for the development of symptoms, which include diarrhea, vomiting, urticaria, dyspnea, and even life-threatening anaphylaxis.

A small group of foods accounts for the majority of allergic reactions (eggs, milk, peanuts, tree nuts, fish, shellfish, soy, and sesame). Although they share symptomatology and immunological mechanisms, these types of food allergies seem to have differences. Peanut allergy, for example, has a lower rate of spontaneous desensitization (approximately 22%) compared with egg and milk allergy (approximately 50%) (5–7). When it comes to severity, allergic reaction to peanuts is more associated with hospitalization due to anaphylaxis (8). Moreover, these foods have different numbers of allergens, distinct allergen structures, and singular food matrix. All of these might account for singularities of different types of food allergies, which is an interesting field to explore.

Experimental models are an effective tool to study food allergies, as they replicate some of the key immunological mechanisms associated with sensitization and allergic reaction in humans. Because the possibilities to create food allergy experimental models are almost limitless, there is an immense variability of protocols in the literature. Based on this, it is difficult to make general assumptions and point out specific features of allergies to different types of food proteins. A good approach to this problem would be standardizing an experimental model in which the only varying factor is the allergenic food.

Our laboratory, along with collaborators, has developed a standardized model to study different types of food allergy. We sensitize mice with a given allergen adsorbed in aluminum hydroxide and challenge them with a drinking solution containing the allergenic food. With this model, we have been able to study allergy to eggs, milk, and shrimp (9–11). Because peanut allergy is one of the most common types of food allergies and has important clinical features, we aimed to develop an experimental model of peanut allergy based on our standardized protocol.

## 2 Materials and methods

### 2.1 Animals

Male BALB/c and C57BL/6 mice aged between 6 and 8 weeks were obtained from the Breeding Animal Facility at Universidade Federal de Minas Gerais (UFMG). They were given chow and water *ad libitum* and were kept in a 12 h light/dark cycle. All procedures were approved by the Ethics Committee in Animal Experimentation at UFMG (license 339/2019).

### 2.2 Peanut protein extract

Peanut protein extract (PPE) was prepared according to Walczyk et al. (12), with minor alterations. Raw peanuts (Yoki) were peeled and crushed into a flour with a blender, and it was defatted by adding hexane (Sigma-Aldrich 32293) (weight/volume, 1:3). The solution was stirred and left in a fume hood until complete precipitation (20–30 min). After discarding the supernatant, the procedure was repeated twice (for a total of three cycles). At the end, the defatted flour was left in the fume hood overnight for complete hexane evaporation.

Defatted peanut flour was diluted in 20 mM Tris buffer (pH 8.5; weight/volume, 1:30) and left on a magnetic homogenizer for 30 min. After sifting the solution, it was centrifuged at 100,000 × *g*, 4°C, for 1 h. Protein supernatant was collected, stored at −80°C, and used for sensitization of mice.

### 2.3 Whole peanut extract

Whole peanut extract (WPE) was prepared for ingestion during the experimental protocol. Non-defatted peanut flour was diluted in potable water (pH 8.5; weight/volume, 1:30) and homogenized for 30 min. After sifting, it was stored at 4°C for up to 72 h.

### 2.4 Protein profile of peanut extracts

The profile of peanut proteins in PPE was assessed by SDS-PAGE. Fifteen micrograms of PPE were loaded onto a 15% acrylamide gel and separated by 150 V for 2 h. Protein bands were stained with Coomassie and identified according to their molecular weight (kDa), as previously described (13).

### 2.5 Sensitization and oral challenge

**Figure 1:**
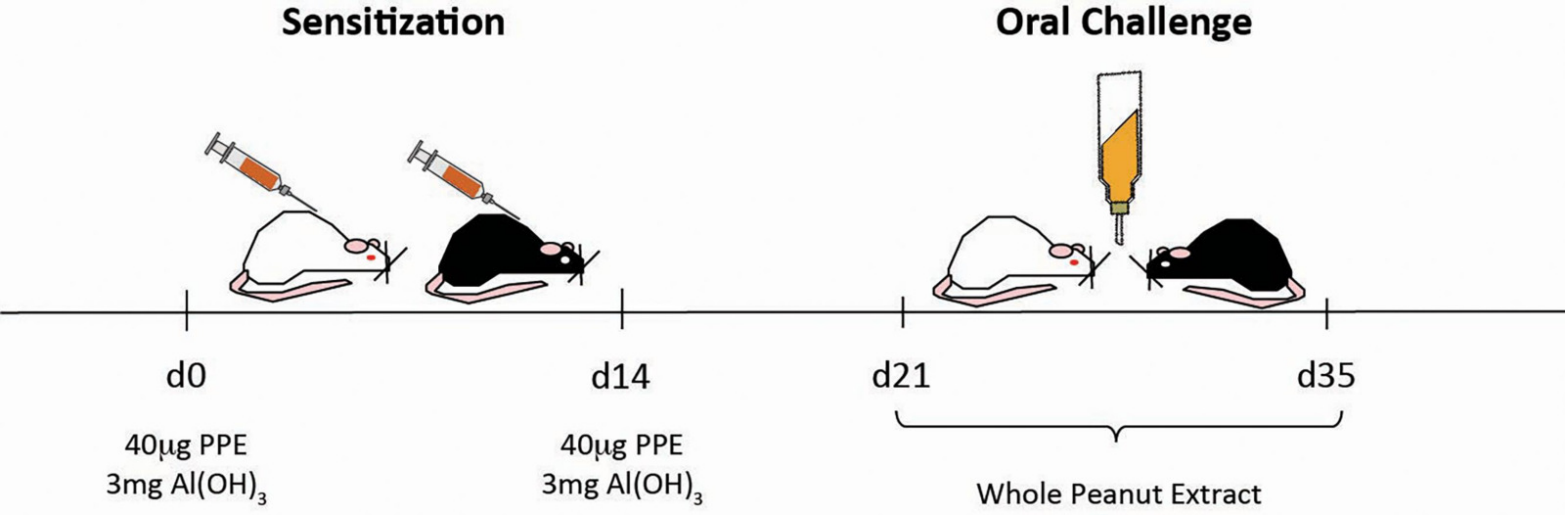
Experimental model of peanut allergy. Young male BALB/c and C57BL/6 mice were sensitized with a subcutaneous injection of 40 µg of PPE adsorbed in 3 mg of Al(OH)_3_ on days 0 and 14. From days 21 to 35, mice were orally challenged with WPE in a drinking bottle as the only option of beverage. Control mice received antigen-free, adjuvant-containing subcutaneous injections and drank WPE during oral challenge, whereas naïve mice did not receive any treatment and drank water.

Young, male BALB/c and C57BL/6 mice were sensitized by a subcutaneous injection of 40 µg PPE adsorbed in 3 mg Al(OH)_3_, diluted to a final volume of 200 µL saline, on days 0 and 14. Control mice received PPE-free, adjuvant-containing injections. From days 21 to 35, drinking bottles containing WPE were offered to sensitized and control mice *ad libitum* as the only option of drinking solution. The content of the bottles was renewed every day. Naïve mice didn't receive injections and drank only water throughout the experimental protocol. The experimental model is represented in Figure 1.

### 2.6 Whole peanut extract ingestion and body weight alteration

During oral challenge, mean WPE ingestion was monitored daily and determined as the difference in drinking bottle's weight after 24 h, which was divided by the number of mice in the cage. An individual mouse drinks approximately 5 mL per day of liquid solution when caged with others, as already described previously (9–11). This method was preferred over daily gavage administration to avoid the stress associated with handling the animals over an extended period (14 days). Body weight alteration was determined as the percentage of body weight change after 14 days of oral challenge.

### 2.7 Gastrointestinal transit time

Control and sensitized mice were individually caged and gavaged with 15 mg of WPE in 6% carmine red (diluted in 0.5% methylcellulose; 300 µL final volume). Each cage was checked for stained fecal pellets after 30, 45, and 55 min. Subsequently, cages were checked every 5 min. Gastrointestinal transit time was determined as the time difference between gavage and stained fecal pellet detection.

### 2.8 Total and anti-PPE antibody detection

At the end of oral challenge, serum samples were collected for anti-PPE antibody detection by ELISA. For specific IgE and IgG1, a 96-well MaxiSorp plate (Thermo Fisher Scientific Nunc 43954) was coated with PPE (6 μg/well in 50μl for IgE and 2 μg/well in 100 μl for IgG1) in sodium carbonate (pH 9.6) overnight at 4°C. After blocking it with 1%BSA PBS for 1 h at room temperature, plates were incubated with pure serum for IgE (2 h at room temperature). For IgG1, an eight-step serial dilution (starting at 1:2,000) was performed for incubation (1 h at 37°C). Plates were then loaded with rat anti-mouse IgE-biotin (SouthernBiotech 1130-08) for 2 h at room temperature or with goat anti-mouse IgG1-HRP (SouthernBiotech 1070-05) for 1 h at 37°C. For IgE only, an extra step was added for streptavidin–HRP incubation (SouthernBiotech 7100-05) for 1 h at room temperature. ABTS (Sigma-Aldrich A1888; 0.5 mg/mL + 1 µL/mL H_2_O_2_ in citrate buffer pH 5.0) was used for the colorimetric reaction. Plates were washed with 0.05% Tween 20 PBS between each step.

For total IgE detection, plates were coated with rat anti-mouse IgE-UNLB (SouthernBiotech 1130-01) overnight at 4°C, and all other steps were the same as described before.

For total and anti-PPE secretory IgA (SIgA), plates were coated with goat anti-mouse Ig (SouthernBiotech 1010-01) or PPE (2 µg/well) in sodium carbonate (pH 9.6) overnight at 4°C. Fecal pellets were homogenized in PBS, 100 mg per 1,000 µL, and centrifuged at 1,200 rpm, 20 min, 4°C. After blocking the plate with 1% BSA PBS for 1 h at room temperature, plates were incubated with fecal supernatant (1:5 for total SIgA and undiluted for anti-PPE SIgA) for 1 h at 37°C. Next, plates were loaded with goat anti-mouse IgA HRP (SouthernBiotech 1040-05) for 1 h at 37°C. ABTS (Sigma-Aldrich A1888; 0.5 mg/mL + 1 µL/mL H_2_O_2_ in citrate buffer pH 5.0) was used for the colorimetric reaction. Plates were washed with 0.05% Tween 20 PBS between each step.

Total IgE and IgA were expressed as protein concentration. Standard curves for total IgE and IgA were used to determine specific IgE and IgA levels, respectively, through heterologous interpolation, and data were expressed as arbitrary units. Specific IgG1 was expressed as the optical density sum of each step of samples' serial dilution serial dilution.

### 2.9 IgA-bound bacteria

Fecal pellets were homogenized in PBS (100 mg per 1,000 µL) and centrifuged at 400 × *g* for 5 min at 4°C. Supernatant was filtered through a 40 µm cell strainer and centrifuged at 8,000 × *g* for 5 min. After discarding the supernatant, bacteria pellet was resuspended by vigorous vortexing and washed in PBS. Bacteria pellets were incubated with purified rat anti-mouse CD16/CD32 (BD Pharmingen 553142) for 20 min at 4°C. After washing with PBS, the bacteria pellets were incubated with anti-mouse IgA PE (eBioscience 12599481) for 30 min at 4°C. IgA-bound bacteria were analyzed by flow cytometry.

### 2.10 Immunoblotting

Peanut proteins (15 µg) were separated in an acrylamide gel (as described in Section 2.4) and transferred to a nitrocellulose membrane. After blocking with 0.3% Tween 20 PBS, the membrane was incubated with pooled serum (1:3,000) from the control and sensitized groups for 1 h at room temperature. Next, incubation with goat anti-mouse IgG1-HRP (SouthernBiotech 1070-05) for 1 h at room temperature, followed by a chemoluminescence reaction, revealed the immunogenic protein bands.

### 2.11 Active and passive cutaneous anaphylaxis

For active cutaneous anaphylaxis, at day 21 of the experimental protocol, mice were anesthetized (80 mg/kg ketamine and 15 mg/kg xylazine in 100 µL saline) and received an intradermal injection of 50 µg PPE in the ear. Immediately after, 200 µL of 0.5% Evan's blue was injected intravenously, and the cutaneous reaction happened for 30 min. After euthanasia, the ears were collected and placed in formamide for 72 h. The amount of dye extravasation was determined by spectrophotometry.

For passive cutaneous anaphylaxis, a pool of undiluted serum (30 µL) from the control and sensitized groups was injected intradermally in the back of naïve mice. After 48 h, these mice were anesthetized (80 mg/kg ketamine and 15 mg/kg xylazine in 100 µL saline) and challenged with an intravenous injection of 300 µg PPE in 0.5% Evan's blue. The cutaneous reaction was measured by the diameter of dye extravasation.

### 2.12 Systemic anaphylaxis

Body temperature of control and sensitized mice was measured with an infrared thermometer. Subsequently, each mouse received an intraperitoneal injection of 500 µg of PPE (diluted in saline, 200 µL final volume). Then, body temperature was reassessed after 15, 30, 60, and 120 min as a measurement of systemic anaphylaxis reaction.

### 2.13 Histological analysis

At the end of the experimental protocol, the proximal jejunum was fixed in 10% formalin, embedded in paraffin, and cut into 5 µm-thick sections. They were stained with hematoxylin and eosin and scanned with a digital microscope scanner. Using CaseViewer 1.4 software, three random sections at 20× magnification (15–20 villi per mouse) were considered for eosinophil, intraepithelial lymphocytes (IELs), and goblet cells count, as well as villus height and crypt depth measurement.

For mast cell count, slides were stained with toluidine blue, tissue area was measured, and total mast cell number was quantified with QuPath software. Data were expressed as the number of mast cells per square millimeter (mm^2^).

### 2.14 Statistical analysis

Normal distribution of samples was confirmed by the Shapiro–Wilk test. One-way analysis of variance (ANOVA) followed by Tukey's test, or Student’s *t*-test, was used for significance detection between groups (*p* ≤ 0.05). All data analysis was performed with GraphPad Prism® 8.1.1.

## 3 Results

### 3.1 Sensitized mice developed specific antibody response against the peanut protein extract

**Figure 2:**
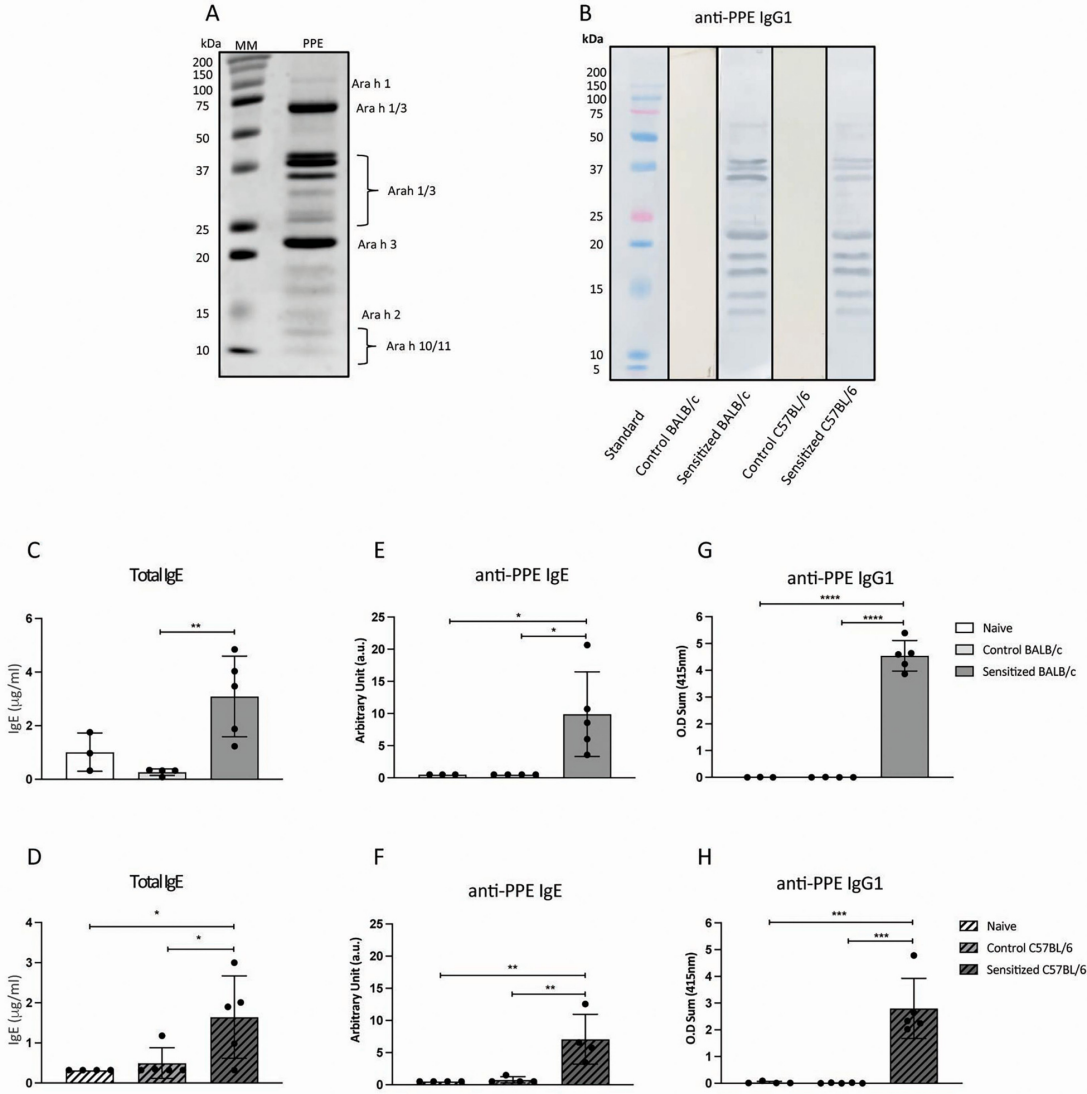
Antibody response of sensitized mice after oral challenge. **(A)** Gel electrophoresis of peanut proteins extracted in Tris buffer. The definition of peanut allergens was based on the molecular weight of these proteins reported by Walczyk et al. (13). **(B)** Western blot of PPE incubated with pooled sera from the sensitized and control groups, from both strains, at the end of the experimental protocol. Young male BALB/c (*n* = 5) and C57BL/6 (*n* = 5) mice were sensitized with a subcutaneous injection of 40 µg of PPE adsorbed in 3 mg of Al(OH)_3_ on days 0 and 14. Control mice (*n* = 4 BALB/c; *n* = 5 C57BL/6) received antigen-free injections. From days 21 to 35, mice were orally challenged with WPE in a drinking bottle as the only option of beverage. Naïve mice (*n* = 3 BALB/c; *n* = 4 C57BL/6) did not receive any treatment. At the end of the protocol, serum was collected for the measurement of **(C)** total IgE, **(E)** anti-PPE IgE, and **(G)** anti-PPE IgG1 from BALB/c mice, as well as **(D)** total IgE, **(F)** anti-PPE IgE, and **(H)** anti-PPE IgG1 from C57BL/6 mice. Data are expressed in mean ± standard deviation. *p ≤ 0.05; **p ≤ 0.01; ***p ≤ 0.001; ***p ≤ 0.0001. Representative of three experimental replicates.

The extraction of proteins from defatted peanut flour in 20 mM Tris buffer (PPE) led to the detection of five major bands (from ∼25 to ∼75 kDa) that correspond to *Arachis hypogea* (Ara h) 1 and Ara h 3 (Figure 2A). Other allergens of lower molecular weight, such as Ara h 2 (15 kDa), as well as Ara h 10 and Ara h 11 (∼10 kDa), were also identified. The same allergen with different molecular weight reveals either posttranslational alterations or subunits of the same protein (13). To determine which peanut allergens from PPE elicited a humoral response, we performed a Western blot with pooled serum from sensitized mice. There was a clear interaction between IgG1 antibodies and bands corresponding to Ara h 1 and Ara h 3 (Figure 2B). Although less evident, we also detected a reaction of IgG1 antibodies with Ara h 2. These results show that nearly all allergens in PPE were immunogenic, as it was expected, since Ara h 1, Ara h 2, and Ara h 3 are major allergens associated with peanut allergy.

IgE is the most important antibody isotype in food allergy because it binds with high affinity to mast cells via FcɛRI and leads to their degranulation by cross-linking with allergens. After oral challenge, sensitized BALB/c and C57BL/6 mice had higher levels of total and specific IgE (Figures 2C–F) in their sera than the control and naïve groups. Thus, WPE ingestion by sensitized, but not by control mice, led to an increase in IgE response, a hallmark of food allergy.

Although IgG effects on food allergy remain controversial, IgG1 seems to be a good biomarker for peanut allergy (14). In this experimental model, WPE ingestion resulted in high serum anti-PPE IgG1 levels in sensitized mice from both strains (Figures 2G,H), but no significant levels of this antibody were found in the control and naïve groups. Thus, not only do these mice produce specific IgE, but they also have high titers of serum-specific IgG1, an important aspect of peanut allergy in humans.

### 3.2 Sensitization led to mast cell degranulation upon allergen exposure in both BALB/c and C57BL/6 mice

**Figure 3:**
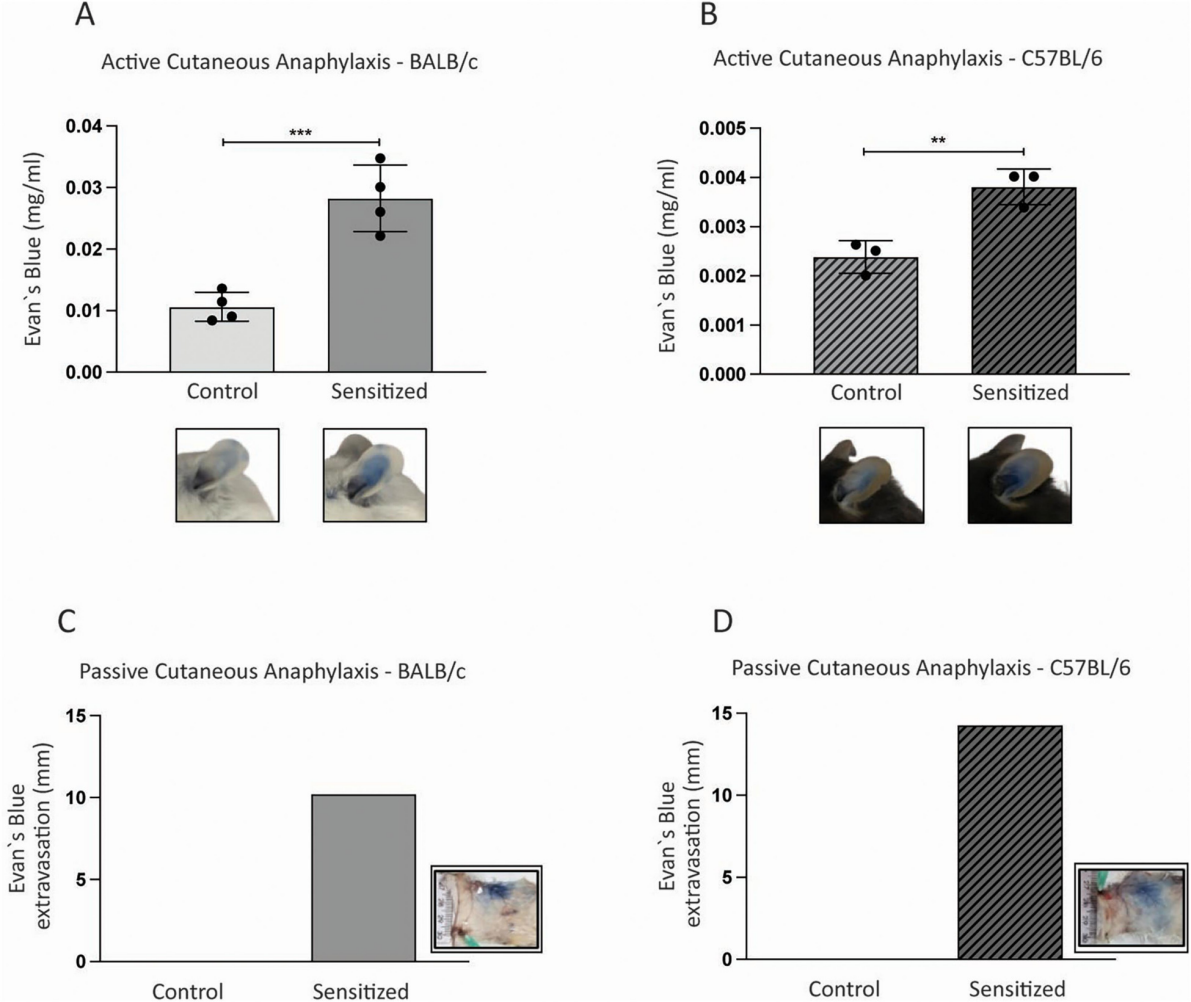
Mast cell degranulation upon exposure to peanut protein extract. **(A)** Young male BALB/c (*n* = 4) and **(B)** C57BL/6 (*n* = 3) mice were sensitized with a subcutaneous injection of 40 µg of PPE adsorbed in 3 mg of Al(OH)_3_ on days 0 and 14. Control mice (*n* = 4 BALB/c; *n* = 3 C57BL/6) received antigen-free injections. On day 21, mice received an intravenous injection of Evan's blue (0.5%) and were subsequently challenged with an intradermal injection (ear) containing 50 µg of PPE. Evan's blue extravasation was an indirect measure of mast cell degranulation. In a different set of experiments, after oral challenge, 30 µL of undiluted pooled sera from the **(C)** BALB/c and **(D)** C57BL/6 groups was transferred to naïve mice (*n* = 3 per group) through an intradermic injection in the back. After 48 h, these mice received an intravenous injection containing 300 µg of PPE diluted in 0.5% Evan's blue. Dye's extravasation at the site of serum injection was an indirect measure of mast cell degranulation. Data are expressed in mean ± standard deviation. *p ≤ 0.05; **p ≤ 0.01; ***p ≤ 0.001; ***p ≤ 0.0001. One experimental replicate.

Mast cell degranulation is key to the inflammatory response in food allergy. These cells release an array of mediators, such as histamine, prostaglandins, and leukotrienes, which trigger classical allergic symptoms associated with vascular permeability and smooth muscle constriction (4). Thus, we assessed mast cell degranulation in this model. First, at day 21, right after sensitization, we performed an active cutaneous anaphylaxis test by giving mice an intradermal injection of PPE in the ear. As expected, sensitized BALB/c and C57BL/6 mice had greater vascular leakage, an indirect measure of mast cell degranulation, than the control groups (Figures 3A,B). This indicates that mast cells were already sensitized with specific IgE prior to WPE ingestion and were therefore responsive to peanut allergens.

To further determine the relationship between specific antibodies and mast cell degranulation, we took a slightly different approach. After oral challenge, we transferred serum from the sensitized and control groups to naïve mice, and after 48 h, we challenged them. Mice that received serum from the sensitized BALB/c and C57BL/6 groups presented with vascular leakage after systemic PPE challenge, unlike mice that received serum from the control groups (Figures 3C,D). Therefore, not only could we detect anti-PPE IgE in the serum, but we also confirmed that it was able to bind to the surface of mast cells in naïve mice and lead to degranulation upon peanut allergen exposure.

### 3.3 Sensitized mice presented inflammatory alterations in the proximal jejunum after oral challenge

**Figure 4:**
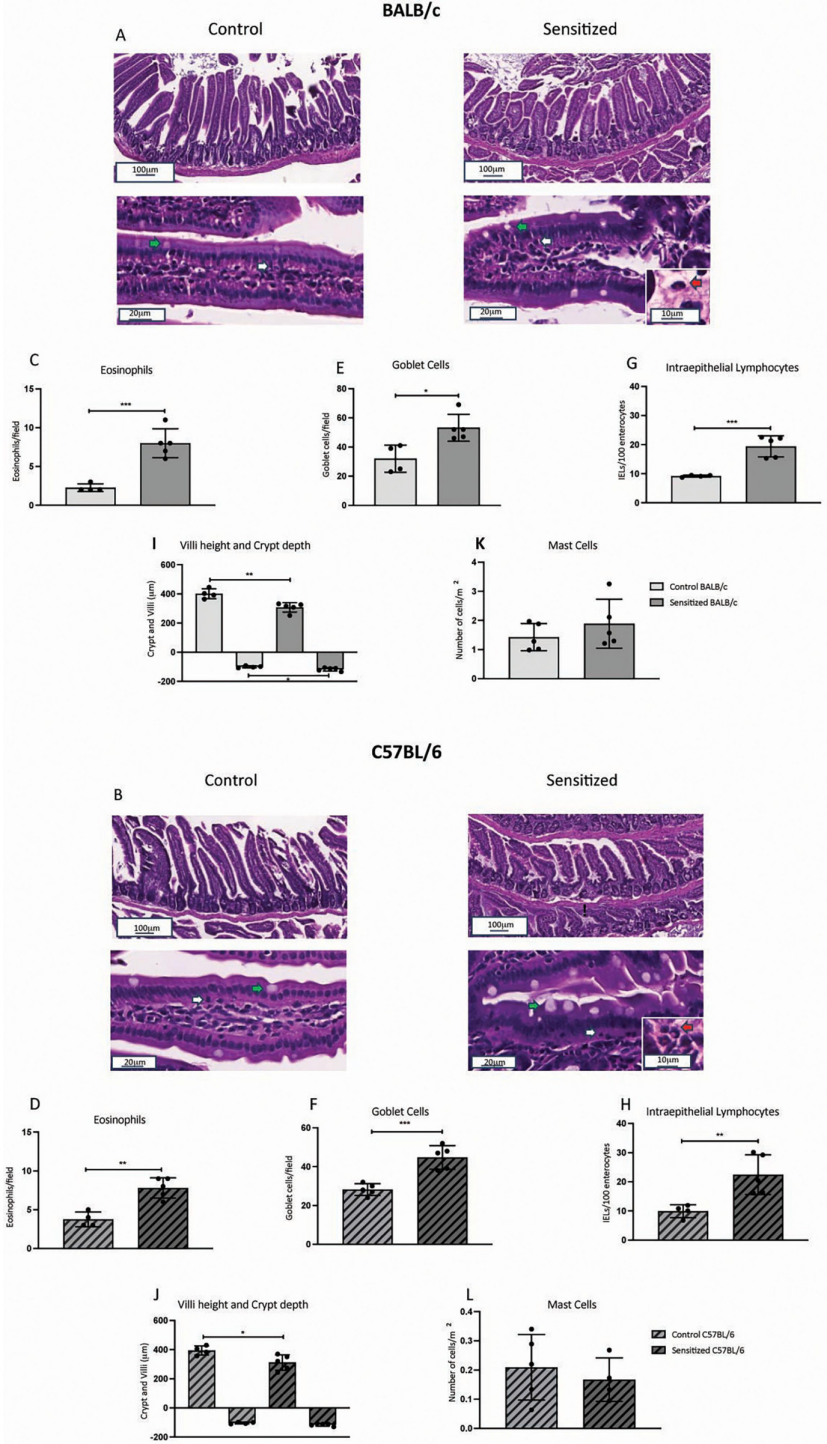
Leukocyte infiltration and morphometric changes in the proximal jejunum of sensitized mice after oral challenge. Young male BALB/c (*n* = 5) and C57BL/6 (*n* = 5) mice were sensitized with a subcutaneous injection of 40 µg of PPE adsorbed in 3 mg of Al(OH)_3_ on days 0 and 14. Control mice (*n* = 4 BALB/c; *n* = 4 C57BL/6) received antigen-free injections. From days 21 to 35, mice were orally challenged with WPE in a drinking bottle as the only option of beverage. At the end of the protocol, the proximal jejunum was collected for histological analysis. **(A,B)** Representative figures of the intestinal mucosa of the control and sensitized BALB/c groups and control and sensitized C57BL/6 groups, respectively. The green arrows show goblet cells, white arrows show IELs, and red arrows show eosinophils. Eosinophil count in **(C)** BALB/c and **(D)** C57BL/6 mice. Goblet cells count in **(E)** BALB/c and **(F)** C57BL/6 mice. IEL count in **(G)** BALB/c and **(H)** C57BL/6 mice. Villus height and crypt depth in **(I)** BALB/c and **(J)** C57BL/6 mice. Mast cell count in **(K)** BALB/c and **(L)** C57BL/6 mice. Data are expressed in mean ± standard deviation. *p ≤ 0.05; **p ≤ 0.01; ***p ≤ 0.001; ***p ≤ 0.0001. Representative of two experimental replicates.

Type 2 immune responses, such as allergies, are closely associated with features such as eosinophil infiltration and goblet cell hyperplasia (15). As illustrated in Figures 4A and B, histological analysis of the proximal jejunum showed higher counts of eosinophils, goblet cells, and IELs in sensitized BALB/c and C57BL/6 mice after oral challenge (Figures 4C–H). These mice also presented smaller villi, and for BALB/c mice only, the sensitized group had larger crypts than the control group. Mast cell count, however, revealed no significant difference between the control and sensitized groups in both strains (Figures 4K,L). All the inflammatory and morphological changes indicate a hostile environment in the intestinal lumen due to the presence of peanut allergens. Thus, this model replicates classical inflammatory alterations associated with food allergy in the intestinal mucosa.

### 3.4 Sensitized BALB/c mice presented increased anti-PPE SIgA production and reduced SIgA reactivity to intestinal bacteria

**Figure 5:**
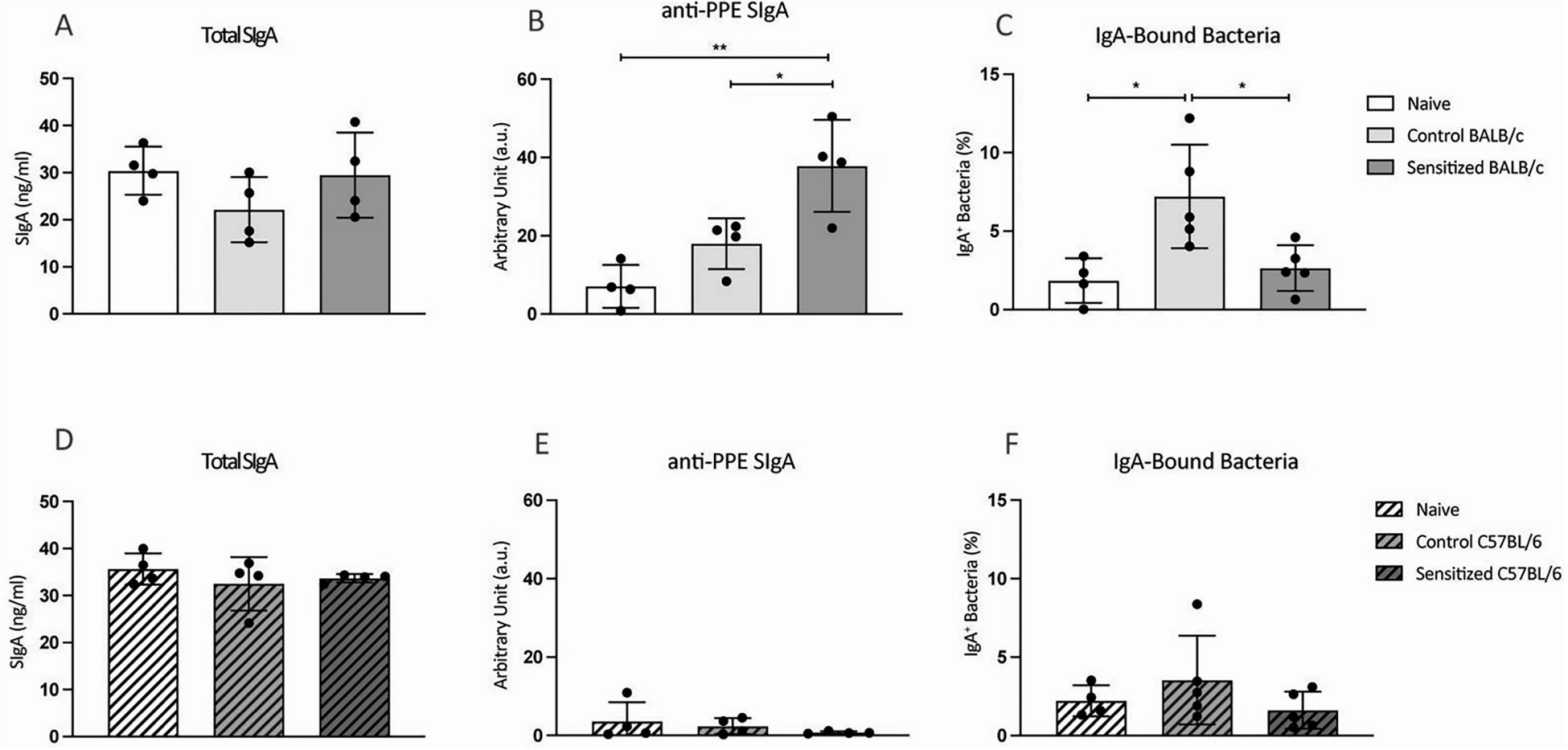
Fecal SIgA response in sensitized mice after oral challenge. Young male BALB/c (*n* = 4) and C57BL/6 (*n* = 4) mice were sensitized with a subcutaneous injection of 40 µg of PPE adsorbed in 3 mg of Al(OH)_3_ on days 0 and 14. Control mice (*n* = 4 BALB/c; *n* = 4 C57BL/6) received antigen-free injections. From days 21 to 35, mice were orally challenged with WPE in a drinking bottle as the only option of beverage. Naïve mice (*n* = 4 BALB/c; *n* = 4 C57BL/6) did not receive any treatment. At the end of the protocol, fecal samples were collected for total and specific SIgA analysis, as well as bacteria-bound IgA. **(A)** Total and **(B)** Anti-PEE fecal SIgA in BALB/c mice. **(C)** Frequency of IgA-bound bacteria in BALB/c mice. **(D)** Total and **(E)** Anti-PEE fecal SIgA in C57BL/6 mice. **(F)** Frequency of IgA-bound bacteria in C57BL/6 mice. Data are expressed in mean ± standard deviation. *p ≤ 0.05; **p ≤ 0.01; ***p ≤ 0.001; ***p ≤ 0.0001. Representative of two experimental replicates.

Secretory IgA (SIgA) is the most abundant antibody isotype in the intestinal mucosa, where it binds both microbial and food antigens (16, 17). After oral challenge, sensitized BALB/c mice presented higher levels of anti-PPE SIgA than the control groups (Figure 5A). Although there was no change in total SIgA, its reactivity to intestinal bacteria was diminished in sensitized BALB/c mice (Figure 5B,C). For C57BL/6 mice, however, fecal anti-PPE SIgA was very low in all groups, with no difference among them (Figure 5D). Similarly, there were no changes in total SIgA production and reactivity to intestinal bacteria (Figure 5E,F). Thus, in this model, intestinal response to WPE ingestion associates with changes in SIgA reactivity in sensitized BALB/c, but not C57BL/6, mice.

### 3.5 Sensitized BALB/c and C57BL/6 mice presented distinct local and systemic allergic reactions

**Figure 6:**
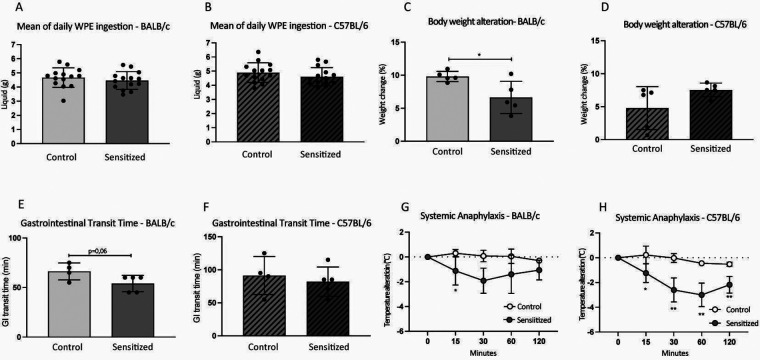
Clinical features during oral challenge. Young male BALB/c (*n* = 5) and C57BL/6 (*n* = 5) mice were sensitized with a subcutaneous injection of 40 µg of PPE adsorbed in 3 mg of Al(OH)_3_ on days 0 and 14. Control mice (*n* = 5 BALB/c; *n* = 5 C57BL/6) received antigen-free injections. From days 21 to 35, mice were orally challenged with WPE in a drinking bottle as the only option of beverage. **(A,B)** WPE ingestion. **(C,D)** Percentage of body weight alteration after oral challenge. **(E,F)** After 7 days of oral challenge (day 28), sensitized BALB/c (*n* = 4) and C57BL/6 (*n* = 4) mice were gavaged with 15 mg of PPE for the determination of gastrointestinal tract transit time. **(G,H)** After 7 days of oral challenge (day 28), sensitized BALB/c (*n* = 4) and C57BL/6 (*n* = 4) mice were challenged with an intraperitoneal injection of 500 µg of PPE to determine systemic anaphylaxis. Data are expressed in mean ± standard deviation. *p ≤ 0.05; **p ≤ 0.01; ***p ≤ 0.001; ***p ≤ 0.0001. One experimental replicate.

According to murine studies with similar protocols of food allergy to egg and milk, sensitized mice may present changes in the ingestion of solutions containing the allergen to which they were sensitized, as well as weight loss during oral challenge (9, 10). In this model, however, sensitized mice from both strains ingested the same amount of WPE compared with the control groups (Figures 6A,B). Moreover, sensitized mice did not present weight loss after oral challenge (Figures 6C,D). Despite the lack of changes in these parameters, sensitized BALB/c, but not C57BL/6, mice tended to increase gastrointestinal transit time after PPE gavage compared with the control group (Figures 6E,F). However, upon systemic challenge with PPE, sensitized BALB/c mice presented only a moderate drop in body temperature compared with the control group (Figure 6G), while a strong change was observed in sensitized C57BL/6 mice (Figure 6H), indicating an anaphylactic reaction. Thus, sensitized mice did not present with differences in allergen ingestion and weight change during oral challenge; however, they presented clinical features associated with food allergy, with singularities according to mouse strain.

## 4 Discussion

The present study aimed to develop an experimental model of peanut allergy that enables both detailed investigation of the disease and direct comparison with other food allergy models developed by our group (9–11).

Sixteen peanut allergens have been identified (18). Out of these, Ara h 1, Ara h 2, and Ara h 3 seem to be major ones because a high percentage of patients are sensitized to one or more of them (18, 19). Through gel electrophoresis, we detected all three of these proteins in our PPE. As expected, sensitized BALB/c and C57BL/6 mice mounted a humoral response against Ara h 1, Ara h 2, and Ara h 3. Thus, our model successfully replicated an important sensitization pattern in the clinical setting.

IgE production is a hallmark of food allergy. Despite limitations, serum IgE levels can be used as a predictor of positive oral challenge and, therefore, clinical allergy (20, 21). In experimental models, specific serum IgE is used as a major indicator of successful sensitization and tends to increase after oral challenge (9, 11, 22). In this model, sensitized BALB/c and C57BL/6 mice presented higher levels of total and PPE-specific IgE than the control groups after WPE ingestion. As expected, WPE ingestion alone did not cause changes in IgE production.

The essential role of IgE in food allergy has much to do with its ability to bind to the surface of mast cells and rapidly induce their degranulation upon allergen exposure. Thus, mast cells play a major role in IgE-mediated food allergies. Besides being present at mucosal surfaces, these cells also populate the skin. This is why the skin prick test is widely used in the clinical setting for food allergy diagnosis, as it is associated with positive oral challenge and allergy persistence as children age (3, 21). Here, we showed that sensitized mice, prior to oral challenge, had greater mast cell activation upon PPE ear-exposure than the control groups, regardless of mouse strain. This points to a successful sensitization, because mast cells were already bound to IgE prior to oral exposure to WPE. Moreover, we confirmed this was indeed antibody-mediated through a passive cutaneous anaphylaxis test using postoral challenge serum from the sensitized BALB/c and C57BL/6 groups. Thus, this model of peanut allergy involved production of specific IgE, which was capable of inducing mast cell degranulation, resembling a key aspect of clinical peanut allergy.

IgG1 is also involved in allergic immune response. It can signal through Fc*γ*RIII in basophils and other cell types to induce anaphylaxis in the context of food and non-food antigens (23–25). IgG1 is also crucial for high-affinity IgE responses since IgG1-producing B cells can undergo affinity maturation and turn into IgE-producing B cells (26). Moreover, IgG1 is a potential biomarker of peanut sensitization status in humans (14). In our model, sensitization followed by oral challenge led to high titers of anti-PPE IgG1 in the serum of BALB/c and C57BL/6 mice. Although we have not determined the functional properties of IgG1 in the present study, we observed that C57BL/6 had high levels of serum-specific IgG1 and an increased susceptibility to systemic anaphylaxis. Thus, our findings on the humoral response to PPE align with aspects of clinical peanut allergy.

Little is known about the role of SIgA in the immune response to food antigens. Although it would be reasonable to assume that food-specific SIgA is essential to food tolerance, Liu et al. (27) showed that it does not correlate with oral tolerance nor predict protection from food allergy development in humans. In mice, Nakajima et al. (16) found that the majority of naïve mice that ingested a peanut diet for 42 days did not produce detectable levels of peanut-specific SIgA. However, upon oral sensitization with cholera toxin (CT), these mice start producing high levels of peanut-specific SIgA. Our data are in line with these findings, since BALB/c naïve and control mice produced low levels of PPE-reactive SIgA, while an increase was seen in the sensitized group. Interestingly, very low levels of PPE-reactive SIgA were detected in the feces of C57BL/6 mice from all groups. Concerning non-sensitized mice, our data are similar to the findings of Nakajima et al. (16), who used the same mouse strain (C57BL/6) in their experiments. They, however, detected anti-peanut SIgA in mice sensitized with CT. This suggests that, unlike BALB/c mice, C57BL/6 mice require a mucosal route of sensitization for the induction of peanut-specific SIgA response. Moreover, since IgA production requires the orchestrated action of TGF-ɩ*σ*, IL-4, and IL-5 (28–30), which are cytokines antagonized by INF*γ* (31, 32), it is possible that C57BL/6, a strain with a predominant Th1 profile of immune response, requires higher stimulation by Th2-adjuvant inducers to produce detectable levels of specific SIgA in the intestinal lumen.

Contrary to anti-PPE SIgA, we did not detect changes in total SIgA upon sensitization followed by oral challenge. However, we found a decrease in IgA-bound bacteria in the feces of sensitized BALB/c mice compared with the control group. It has been reported that SIgA in the intestine is polyreactive and, therefore, binds a broad spectrum of microbiota (33). This helps contain commensal microbiota and prevent undesirable contact between pathogenic microorganisms and the lamina propria. The decrease in IgA-bound bacteria in sensitized mice may reflect a deviation of reactivity from microbiota toward peanut proteins. Thus, the polyreactive IgA compartment may be taken over by peanut protein-reactive IgA, which might impair the relationship between the intestinal mucosa and commensal bacteria in allergic BALB/c mice.

WPE ingestion by sensitized BALB/c and C57BL/6 mice caused intestinal mucosa inflammation, evidenced by higher eosinophil and IELs count. Eosinophils depend on IL-5 for survival and express the high-affinity receptor for IgE. When activated, they can release an array of proteases that cause damage to the surrounding tissue (34). Although little is known about the effects of IELs on food allergy, these cells are essential for the maintenance of immunological tolerance and surveillance, as well as epithelial barrier integrity in the intestinal mucosa (35). They can also be highly cytotoxic upon activation. Thus, increased numbers of eosinophils and IELs might be damaging to the intestinal mucosa, which probably is a result of allergen-driven changes in the relationship between mucosal immune compartments (IELs and lamina propria) and intestinal lumen. Alternatively, increased numbers of IELs may represent a compensatory mechanism involved in protection against further inflammatory damage to the epithelium.

We also observed an increase in goblet cell count and intestinal crypt size. Goblet cells are known for producing mucus, which attaches to the epithelium and confers protection from damaging molecules and pathogenic microbes (36). Intestinal crypts house stem cells that differentiate and give rise to epithelial cell subsets that lie in the villi (37). Based on their functions, more mucus-producing cells and larger stem cell compartments seem to be protective mechanisms induced to increase the epithelial barrier and renew damaged epithelial cells in an inflammatory context triggered by peanut allergens.

Some studies have reported that susceptibility to peanut allergy differs according to mouse strain. With two distinct methodological approaches, Smit et al. (38) and Paolucci et al. (39) showed C3H mice are more susceptible to systemic anaphylaxis than BALB/c and C57BL/6 mice. The former group also found that the C57BL/6 strain is more prone than the BALB/c strain to decreasing body temperature after challenge with peanut extract. Although we did not intend to compare peanut allergy susceptibility between BALB/c and C57BL/6 mice in our model, we similarly saw that the latter presented a stronger anaphylactic reaction, which is probably related to the engagement of IgG1 in addition to IgE (38). However, when gastrointestinal transit time upon challenge was assessed, sensitized C57BL/6, but not BALB/c, mice failed to present a faster transit time compared with the control group. This is probably due to the presence of the *Dpep1* (*dipeptidase 1*) gene in C57BL/6 mice that confers resistance to orally induced allergic reactions such as anaphylaxis (40). Thus, while systemic anaphylaxis is clearly seen in C57BL/6 mice, local intestinal responses, such as an increase in transit time, seem to be more prominent in BALB/c mice, which is further evidenced by differences in SIgA production. However, since sensitized mice clearly differed from the control groups in both strains in terms of clinical and immunological features, our food allergy model was reproducible in the BALB/c and C57BL/6 strains of mice. This is an advantage of the model since it can be applied to either one depending on local availability and scientific questions asked.

Experimental models of peanut allergy are often based on sensitization through the oral route with CT, a potent mucosal adjuvant (38, 41–43). After multiple gavages, with or without boosters, a challenge is given for anaphylaxis assessment. The mice develop classical features of sensitization and allergic response, such as specific IgE production, mast cell degranulation, and anaphylaxis. It is important to mention that the intestinal mucosa damage and local immune response in this context are due not only to peanut but also to CT. This toxin is capable of disrupting the intestinal epithelium and inducing both innate and adaptive immune responses (44, 45). In our model, we use a less aggressive approach to sensitize and challenge mice and still find immunological and clinical alterations associated with food allergy without extra adjuvant effects. Moreover, in our model, antigen delivery is done through a drinking bottle rather than gavage. We chose this approach because ingestion of the allergen allows its passage through the whole gastrointestinal tract, which better represents what happens naturally; it is much less stressful to the animals than subsequent gavages for 14 days, and having the allergen available in the drinking bottle allows mice to choose when and how much to ingest, enabling the assessment of the relationship between mice and the food to which they were sensitized. Lastly, although allergen delivery by gavage guarantees each mouse received the same dose, we have seen that, when individually caged, each mouse drinks comparable amounts of WPE within the same group (data not shown). Moreover, and most importantly, anti-IgG1 and anti-IgE titers are also comparable within the sensitized groups, indicating similar allergen dose delivery.

Lastly, this model of peanut allergy was based on the standardized food allergy protocol from our research group. We use alum hydroxide as an adjuvant to deviate the immune response from tolerance to type 2 immunity toward the chosen allergen. This effect seems to be associated with antigen-presenting cell activation in the presence of IL-4 (46). This explains why, in our models, association of ovalbumin, *β*-lactoglobulin, and shrimp and peanut extracts with alum hydroxide efficiently leads to the production of specific IgE and IgG1 (9–11). In all models previously published, sensitized and challenged mice presented with parameters of intestinal mucosa inflammation, such as increased eosinophil count and altered villi/crypt size (9–11). In this study, we also observed important signs of inflammation in the intestinal mucosa after challenging sensitized mice. Moreover, similar to the model of shrimp allergy, specific IgE was able to bind to mast cells and induce local anaphylaxis in the skin (11). Altogether, our model of peanut allergy was able to replicate important features of food allergy pathogenesis found in our previous models with distinct food allergens.

## 5 Conclusion

In this study, sensitization to PPE followed by 14 days of oral challenge with WPE was able to induce antibody, mast cell, mucosal and clinical responses associated with food allergy in both BALB/c and C57BL/6 mice. Thus, our model is suitable for studying the immunopathology and treatment of peanut allergy and will allow us to study the singularities of different types of food allergies based on the standardized protocol developed by our research group.

## Data Availability

The original contributions presented in the study are included in the article/Supplementary Material; further inquiries can be directed to the corresponding author.
